# The effect of individual counseling based on the GATHER principles on perceived stress and empowerment of the mothers with high-risk pregnancies: an experimental study

**DOI:** 10.1186/s12888-022-04047-2

**Published:** 2022-06-13

**Authors:** Sahar Aliabadi, Arezoo Shayan, Mansoureh Refaei, Leili Tapak, Latif Moradveisi

**Affiliations:** 1 Department of Mother and Child Health, Mother and Child Care Research Center, School of Nursing and Midwifery, Hamadan University of medical sciences, Hamadan, Iran; 2 Department of Midwifery and Reproductive Health, Mother and Child Care Research Center, School of Nursing and Midwifery, Hamadan University of medical sciences, Hamadan, Iran; 3 Department of Biostatistics, School of Public Health, Modeling of Noncommunicable Diseases Research Center, Hamadan University of medical sciences, Hamadan, Iran; 4 Research Center for Behavioral Disorders and Substance Abuse, Hamadan University of Medical Sciences, Hamadan, Iran

**Keywords:** Counseling, Empowerment, Stress, High-risk pregnancy

## Abstract

**Background:**

High-risk pregnancy causes different responses, including negative emotions, feelings of vulnerability and psychological stress in the mother. The aim of this study was to investigate the effect of individual counseling on the empowerment and the perceived stress of high-risk pregnant mothers.

**Methods:**

This study was a two-group experimental study. The study was performed on 82 high-risk pregnant women hospitalized in Fatemieh Hospital in Hamadan, Iran. Inclusion criteria were high-risk pregnancy, being literate, gestational age 24 to 36 weeks. The samples were divided into experimental and control groups using randomized block design. Data were collected using Cohen’s perceived stress scale and Kameda empowerment questionnaires. For the experimental group, four sessions of individual counseling according to GATHER principles (Greet, Ask, Tell, Help, Explain, and Return) were performed for 45–60 minutes for two consecutive weeks. SPSS 25 software was used for data analysis.

**Results:**

The mean score of the perceived stress after the intervention in the control and experimental groups were 27.07(5.80) and 25.30(4.95), respectively (*P* = 0.097). There was a substantial difference in the mean score of empowerment 84.76)9.14) and 88.75 (6.17) (*P* < 0.001) and different dimensions of empowerment (self-efficacy, Future image, self-esteem, Support and assurance from others) between the control and intervention groups after the intervention.

**Conclusions:**

The findings of this study indicate individual counseling is effective in empowering the mothers with high-risk pregnancy but has no significant effect on their perceived stress.

## Background

It is considered a high-risk pregnancy when, due to the presence of one or more proven risk factors, the probability of an adverse outcome for the mother or fetus is greater than the risk for normal pregnant women [1]. Physical and social factors and some problems during current or previous pregnancy make a pregnancy high-risk [2].. The prevalence of high-risk pregnancies is estimated at 22% [3]. One study in Iran reported a prevalence of high-risk pregnancies of 63.5% [4], and another reports 76.4% [5].

High-risk pregnancies are associated with severe complications and lead to high medical, hospital, and rehabilitation costs [6]. Adverse midwifery and neonatal outcomes such as low birth weight, preterm birth are common in these pregnancies [7]. These women are 4.2 times more likely to have severe maternal complications during childbirth [8].

Labelling pregnancy as a high-risk one causes different responses, including negative emotions, feelings of vulnerability and psychological stress [6]. Stress and anxiety can cause problems for both mother and the fetus, especially in women who cannot cope [9]. Studies have shown maternal stress affects the baby’s neurological development, negative emotion, difficult mood and psychiatric disorders [10], anthropometric indices [11], risk of preterm birth and low birth weight [12]. Physical, emotional, cultural and spiritual care and support by health care providers are required to enhance maternal and fetal health during a high-risk pregnancy [6].

Decreased self-esteem and increased anxiety are associated with decreased empowerment of pregnant women [13]. Improving the health status of pregnant women and empowering them is one of the important components of health care [14]. Women’s empowerment in pregnancy can lead to a sense of satisfaction from birth and a sense of success, increased self-esteem, emotional welfare, sense of control over choices and decision making, enhancing the empowerment of self-defense and using timely services [15]. Implementing empowerment programs in various dimensions is necessary to enhance the ability of pregnant women to overcome health-related problems [16].

Studies have shown maternal empowerment and stress affect pregnancy outcome, especially in women with high-risk pregnancies. Lecture and cognitive behavioral methods [17] and Benson’s relaxation therapy reduce the stress among mothers with high- risk Pregnancy [18]. One study found empowerment counseling can reduce the anxiety of pregnant women and increase their self-efficacy [19]. Consulting and educating pregnant women can be effective in promoting independence and empowering them to acquire skills to deal with stress and anxiety during pregnancy and childbirth [20]. Counseling is an intervention method without negative impact on mother and fetus and an effective, cheap and feasible method. Since midwives have a close relationship with the pregnant mother, we can utilize the constructive force of them for counseling women during pregnancy [21]. So far, no study has investigated the effect of counseling on empowerment and stress in high-risk pregnant women, so, the present study was conducted to determine the effect of midwifery counseling on the perceived empowerment and stress of women with high-risk pregnancies.

## Methods

This study was a two-group experimental study conducted from May 2021, to February 2022, in the high-risk pregnancy unit of Fatemieh Hospital in Hamadan, in west Iran. This study was performed on pregnant women who were hospitalized in the high-risk pregnancy unit for various reasons (preeclampsia, diabetes, bleeding, amniotic fluid disorder, preterm labor, reduced fetal movement, intrauterine growth restriction). Inclusion criteria were Iranian nationality, being literate,, hospitalization in high-risk pregnancy unit, gestational age 24 to 36 weeks, and exclusion criteria were experience of traumatic events in the last 6 months (death of loved ones, separation from spouse, etc.) and refusal to give informed content.

### Sampling

The following equation was used to calculate the sample size:

$$n=\frac{\ {\left(\ {z}_{1-\frac{\alpha }{2}}\pm {z}_{1-\beta \kern0.75em }\right)}^2\ \left({\sigma}_1^2+{\sigma}_2^2\right)}{{\left({\mu}_1-{\mu}_2\right)}^2}$$

**Fig. 1:**
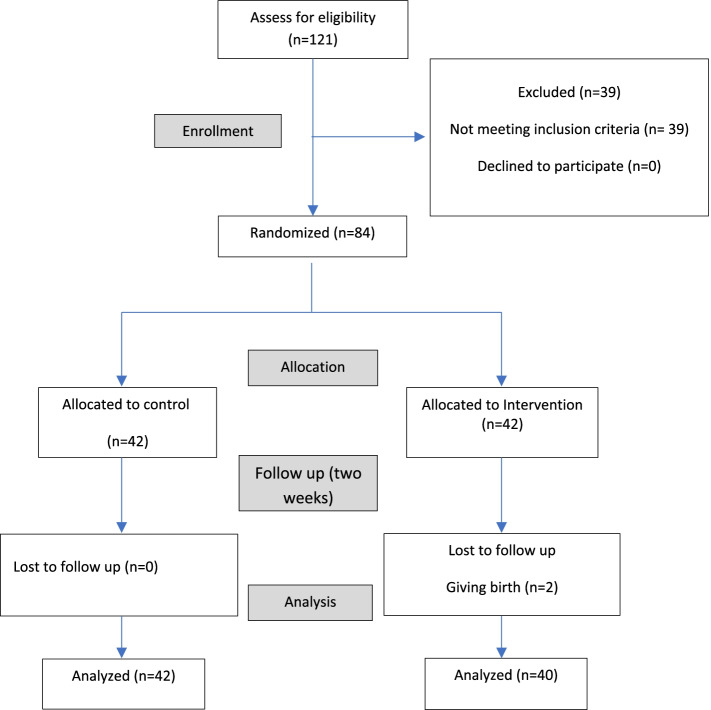
Flow diagram of the participants

Considering the confidence level of the test of 95% (1 − *a* = 0.95) and the test power of 80%, and the common standard deviation of 7.7, the minimum significant difference between the two groups equal to 5 units in terms of stress [22], and 10% possible sample loss, the sample size in each group was estimated to be at least 42 people. Sampling was performed in high-risk pregnancy unit of Fatemieh Hospital in Hamadan. For random allocation, we have used Random allocation software with four permutation blocks. Then the achieved sequence was placed in a matte envelope by someone who was not a member of the research team and labeled from number 1 to 84 and the samples were placed in the intervention and control group according to the numbers. It is noteworthy that the statistical analyst was not aware of the type of groups (control and intervention). Two people from the control group were left out due to giving birth (Fig.1).

### Intervention

First, the researcher introduced herself and after evaluating the people in order to have the inclusion criteria, she explained the purpose of the study and a written consent was taken from them. After completing the questionnaires, individuals were assigned to one of the groups. In this study, the intervention was four sessions of individual counseling in two consecutive weeks between 45 and 60 minutes with pregnant mothers. The consultation was conducted by a master student of counseling in midwifery who had experience working in the high-risk pregnancy unit. The objectives of the sessions were as follows: 1: familiarizing pregnant mother with mental and physiological changes during pregnancy, 2: familiarizing pregnant mother with high-risk pregnancy and how the mother deals with pregnancy risk factors, 3: helping to enhance women’s ability to adapt and accept the existing conditions, and 4: providing information and solutions to reduce stress. The consultation sessions were conducted according to GATHER principles. Which consists of six steps. Step one: welcoming, step two: initiating verbal communication and asking open-ended question on emotions, needs, and concerns. Step three: answering questions and explaining the subject. Step four: helping with decision making. Step five: providing explanation and recommendations for making decisions and actions and step six: explaining the follow- up times. Care was the same in the control and intervention groups, including monitoring the mother’s vital signs, controlling the fetal heart rate, requesting some laboratory tests, and sometimes doing ultrasound. The control group received no amount of counseling and training. The questionnaires were completed again after the fourth session by both groups.

### Data collection tools

Demographic questionnaire, Cohen perceived stress scale and Kameda empowerment questionnaire were used to collect data. Demographic questionnaire had 23 questions regarding the demographics and clinical characteristics of the participants. Cohen perceived stress scale is a 14-question test which is used to determine the general stress within the past month. This questionnaire measures thoughts and emotions on stressful events, control, overcoming, coping with mental pressure and the perceived stress; it is designed to determine people’s stress while facing unforeseeable events and uncontrollable aspects of life. It is graded with five Likert spectra from 0 (never) to 4 (most of the time). The lowest score is zero and the highest score is 56. The higher the score, the greater the perceived stress [23]. The reliability of this questionnaire has been confirmed with 73% Cronbach’s alpha coefficient in Iran [24]. The reliability of the questionnaire in this study was determined using the test-retest (*r* = 94%).

Kameda questionnaire has 27 questions, based on a four choice Likert rating from completely agree to strongly disagree with a score of 1 to 4, with five dimensions, including Self-efficacy (6 questions), Future image (6 questions), self-esteem (7 questions), Support and assurance from others (4 questions) and Joy of an addition person to the family (4 questions), witha minimum score of 27 and a maximum score of 108. A higher score indicates greater empowerment [25]. The validity and reliability of this questionnaire has been confirmed by the content validity and Cronbach’s alpha coefficient (89%) in Iran [26]. The reliability of the questionnaire in this study was determined using the test-retest (r = 88%).

### Data analysis

Mean, standard deviation, frequency, and frequency percentage were used to describe the demographic characteristics and Chi-square test, Fisher test, and analysis of covariance were used to compare the variables in the two groups. The Kolmogorov-Smirnov, and Levin tests were used to evaluate the normality of variables, and homogeneity of variances. Data analysis was performed using SPSS software version 25. In all analyzes, 95% confidence level was considered.

## Results

The mean age of mothers in the control and intervention groups was 31.69(7.42) and 30.73(5.91), respectively. In the control group 40.5% (17) had diploma level education, 85.7% (36) were housewives, 29.3% (12) were hospitalized due to preeclampsia, 26.6% (11) had a history of miscarriage or stillbirth, 9.5% (4) had a history of high-risk pregnancy; These values in the intervention group included the following: 40% (16), 82.5% (33), 42.5% (17), 25% (10), 17.5% (7), respectively. There was no statistically significant difference between the two groups in terms of quantitative (Table 1) and qualitative demographic variables (*p* > 0.05). The two groups were significantly different in terms of participation in childbirth preparation classes (*P* = 0.019) and number of hospitalizations during pregnancy (*P* = 0.022). The effect of significant variables in both groups was adjusted in the analyzes.

**Table 1:** Comparing intervention and control group regarding quantitative variables

Variables	Groups		statistic^a^	*P*-value
	Intervention Mean (SD)	Control Mean (SD)		
Mother’s age (year)	30.73 (5.91)	31.69) 7.42)	0.65	0.51
Partner’s age (year)	36.33 (6.12)	34.71 (8.71)	−0.97	0.33
Pregnancy age	28.47 (2.50)	29.30 (2.90)	1.39	0.16
Number of hispotalization in current pregnancy	1.13 (0.46)	0.85 (0.57)	−2.34	0.022
Number of pregnancies	2.10 (0.96)	2.29 (1.25)	0.778	0.43

^a^ T-test

The mean score of the perceived stress before the intervention in the control and experimental groups were 26.79 (6.22) and 26.60)5.86), respectively; there was no statistically significant difference between the two groups (*P* = 0.561). After the intervention, although the stress score decreased slightly in the intervention group (*P* < 0.001), the difference between the two groups was not significant (*P* = 0.097) (Table 2).

**Table 2:** Adjusted stress and empowerment scores and its dimensions in the control and intervention groups

Variable	Groups		*P*-value*	Effect size
	Control Mean (SD)	Intervention Mean (SD)		
Perceived stress				
Before	26.79 (6.22)	26.60)5.86)	0.561	0.004
After	27.07 (5.80)	25.30 (4.95)	0.097	0.035
*P*-value^**^	0.475	< 0.001		
Empowerment				
Before	82.81 (8.39)	60.08)23.18)	< 0.001	0.282
After	84.76)9.14)	88.75 (6.17)	< 0.001	0.181
*P*-value^**^	0.008	< 0.001		
Self-efficacy				
Before	17.88 (3.52)	14.15 (3.708)	< 0.001	0.213
After	18.62 (3.86)	18.90 (2.28)	< 0.001	0.195
*P*-value^**^	0.005	< 0.001		
Future image				
Before	17.12(2.14)	17.95 (2.39)	0.081	0.052
After	17.42 (2.29)	19.10 (2.01)	0.002	0.119
*P*-value^**^	0.272	< 0.001		
Self-esteem				
Before	21.57(2.89)	17.35 (8.20)	0.006	0.114
After	21.86 (2.99)	22.65 (2.13)	0.008	0.107
*P*-value^**^	0.369	0.002		
Support and assurance from others				
Before	12.50 (1.86)	12.26 (0.32)	0.726	0.002
After	12.40 (2.56)	13.30 (1.95)	0.031	0.059
*P*-value^**^	0.715	< 0.001		
Joy of an addition person to the family				
Before	13.74 (1.25)	13.50 (2.06)	0.585	0.004
After	14.45 (1.64)	14.80 (1.36)	0.325	0.013
*P*-value^**^	0.003	< 0.001		

^*^ Analysis of Covariance, controlled for differences observed before intervention and confounding variables (number of hospitalizations in current pregnancy and participating in childbirth preparation class), ^**^ Paired-t test

The level of the empowerment and all its dimensions increased significantly (*P* < 0.05) after the intervention in the intervention group compared to before the intervention.

The results of covariance analysis for the empowerment and its dimensions showed after the intervention and after adjusting the pre-test effect and confounding variables, the mean score of empowerment (*p* < 0.001), self-efficacy (*p* < 0.001), Future image (*p* = 0.002), self-esteem (*p* = 0.008), Support and assurance from others (*p* = 0.031), had significant differences in the control and intervention groups. The mean score of Joy of an addition person to the family had no significant difference in the groups (*p* = 0.325) (Table 2).

## Discussion

The results show that midwifery counseling for high-risk pregnant mothers has an effective role in increasing their empowerment and in terms of perceived stress, although the stress was slightly reduced, it was not statistically significant.

In this study, the mean score of perceived stress of hospitalized mothers with high-risk pregnancies before the intervention was about 26 in both groups. Naghizadeh et al. showed perceived stress in mothers with high-risk pregnancies is higher than women with low-risk pregnancies [24]. In Karimi et al. study, the perceived stress level of high-risk pregnant women candidates for amniocentesis was 24.76 and about 69.5% had stress above 21.8, which in their study has been interpreted as high stress [22].

In the present study, after the intervention, although the stress was slightly reduced, but this reduction was not significant. Aslani et al. in a study on pregnant women with high perceived stress showed that group education of stress management with a solution-oriented approach, during four weekly sessions, is effective in reducing stress [23]. Golshani et al. showed that the perceived stress score of pregnant women with a history of infertility in the group receiving counseling with a cognitive-behavioral approach is significantly lower than the control group [27]. Khodaparast et al. also showed that educational intervention based on self-regulatory model in women with gestational diabetes reduces perceived stress [28]. Hanan El-Sayed et al. in a study showed that using the Benson relaxation method twice a day for 2 weeks reduced the overall stress score in high-risk pregnant women [18]. In justifying these differences with the present study, the use of different types of intervention may be effective. On the other hand, in comparison with the mentioned studies, this study has been done on high-risk pregnant women who were hospitalized that hospitalization may have affected their stress level. Also in this study, the questionnaires were completed immediately after the consultation of the fourth session, which was related to providing strategies to reduce stress. It seems that women did not have the opportunity to do strategies to reduce stress. Therefore, in subsequent studies, it is recommended that the questionnaires be completed more frequently after the intervention, and more sessions focus on stress.

Ying et al. showed none of the therapeutic interventions including cognitive-behavioral therapy, counseling, mindfulness, stress management and positive reassessment, and other psychological interventions were effective in relieving stress in pregnant women with assisted reproductive techniques [29]. Dargahi et al. also concluded that education has a short-term effect on reducing perceived stress and anxiety in high-risk pregnant women seeking amniocentesis [30]. Therefore, it can be said that stress is a factor that requires several sessions of intervention and expert psychological interventions, and methods such as education alone or a single session of intervention will not be effective in significantly reducing stress in high-risk pregnancies.

This study showed individual counseling for high-risk pregnant mothers has an effective role in increasing their empowerment. Empowerment as an essential psychological factor is related to the physical and mental health of pregnant women [31]. Empowering pregnant mothers by reducing the dangers and warning signs of pregnancy and childbirth and learning relaxation techniques and breathing techniques can reduce the problems of pregnancy [32]. Adeli Gargari et al. showed counseling with empowerment approach is effective in reducing pregnancy anxiety and increasing labor self-efficacy in pregnant women. Counseling with empowerment approach helps the mother to adapt to the physical and mental changes during pregnancy and control her feeling and emotions. This approach increase self-confidence and the skills of gaining the support of others, and independence in decision making [19]. Hooshmandpour et al. Showed midwifery counseling had an effect on the empowerment and self-care of pregnant women. They emphasized the importance of the role of midwives in empowerment counseling [33]. Explaining the importance of the role of midwives in empowering pregnant women compared to other health care providers, Jardim et al. showed the strategies that nurses use to encourage empowerment are considered as scattered methods, and do not provide sufficient knowledge to arrange women’s autonomy [34]. In the study of Hajipour et al. the mean score of empowerment in pregnant women was 78.74, which the highest score was related to self-esteem and the lowest score related to the support and assurance from others, and the Joy of an addition person to the family [26]. In the present study as well, the highest score was related to self-esteem and the lowest score was related to the Joy of an addition person to the family. Explaining this finding can be attributed to factors such as mother’s decreased desire to have children due to the economic and cultural problems, child selfishness, negative experience of pregnancy or previous deliveries.

## Limitations and strengths

Samples of this study were high-risk hospitalized pregnant women that few studies have examined effective strategies to reduce their stress and increase their empowerment. Also, having a control group and providing counseling by a person who had academic education in the field of counseling in midwifery were the strengths of this study. The limitations of this study were the variability of maternal hospital stay and the possibility of preterm delivery. Therefore, to reduce the loss of samples, the study was designed so that counseling sessions were conducted within 2 weeks and the questionnaires were completed immediately, so there was not enough time to better investigate the outcome of the study. It is recommended that counseling sessions be held at shorter intervals and in more sessions for these mothers. Also studding the effect of group counseling or spouse counseling is suggested.

## Conclusion

The results of this study show that individual counseling in high-risk hospitalized pregnant women was effective in terms of their empowerment and its dimensions, but had no effect on perceived stress.

## Data Availability

The datasets used and/or analyzed during the current study are available from the corresponding author on reasonable request.
